# Mixed Support for the Temperature‐Size Rule in Wild Freshwater Fishes

**DOI:** 10.1111/ele.70344

**Published:** 2026-02-18

**Authors:** George C. Brooks, Paul N. Frater, Olaf P. Jensen, Gretchen J. A. Hansen, Craig Paukert, Michael Verhoeven, Lyndsie Wszola, Luoliang Xu, Zachary S. Feiner

**Affiliations:** ^1^ Center for Limnology University of Wisconsin‐Madison Madison Wisconsin USA; ^2^ Department of Fisheries, Wildlife and Conservation Biology University of Minnesota St. Paul Minnesota USA; ^3^ Missouri Cooperative Fish and Wildlife Research Unit, School of Natural Resources, U.S. Geological Survey University of Missouri Columbia Missouri USA; ^4^ Missouri Cooperative Fish and Wildlife Research Unit, School of Natural Resources University of Missouri Columbia Missouri USA; ^5^ Office of Applied Science, Wisconsin Department of Natural Resources Science Operations Center Madison Wisconsin USA

**Keywords:** body size trends, climate change, life‐history trade‐offs, metabolic theory of ecology

## Abstract

The temperature‐size rule states that species living in warmer temperatures will grow faster and mature earlier at smaller sizes. While several studies have documented patterns in average body size consistent with the temperature‐size rule in wild populations, a comprehensive test is lacking. Here, we use age and length data of 1.4 million fish across 7 species from 2704 lakes to quantify temperature‐related variation in growth across ontogeny. Our results show that no species fully conforms to the temperature‐size rule; despite patterns of juvenile growth rate and age at maturity typically aligning with the temperature‐size rule, these changes seldom translate to reduced size at maturity or maximum size. We also found evidence that faster life histories in warmer environments are associated with reduced lifespans. A deeper understanding of how temperature shapes growth in natural systems is needed to accurately predict the effects of global warming on wildlife.

## Graphical abstract

We use age and length data of 1.4 million fish from 2704 lakes to evaluate the temperature‐size rule in wild populations. We found evidence that warmer environments are associated with faster life histories and reduced lifespans, but this seldom translates to smaller maximum body sizes. A deeper understanding of how temperature shapes growth in natural systems is needed to accurately predict the impacts of global warming on wildlife.

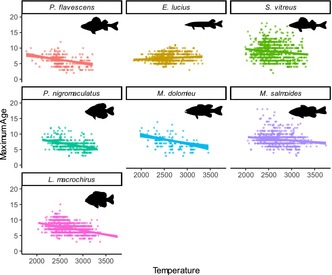

## 1 Introduction

Ecologists have long been fascinated by links between body size and temperature in nature, and early interest on the topic led to the formulation of general patterns, which have since been adopted as ‘rules’ (Allen 1877; Ray 1960). Much of this interest can be attributed to the direct and substantial contributions of body size to individual fitness and ecology, including effects on metabolism (Brown et al. 2004), intra‐ and interspecific competition, predator avoidance (Lawson and Carpenter 2014), abundance (White et al. 2007), and reproduction (Barneche et al. 2018).

The temperature‐size rule is one general pattern that has emerged specifically for ectotherms (Atkinson 1994). The temperature‐size rule states that organisms living in warmer temperatures will (1) grow faster when they are young, (2) mature at an earlier age, and (3) mature at a smaller size. These effects have been observed in 80% of laboratory studies across multiple, diverse taxa including plants, animals, protozoa, and bacteria (Atkinson 1994; Forster et al. 2012). For species with determinate growth, these effects will culminate in smaller maximum body sizes at higher temperatures. Many authors have extrapolated the original temperature‐size rule to make predictions about how maximum body size in species with indeterminate growth will be influenced by temperature (Audzijonyte et al. 2025), and the notion that species might ‘shrink’ with rising temperatures has generated considerable interest considering global warming trends.

The temperature‐size rule has garnered much attention in the study of fisheries affected by climate change owing to its potential to predict and explain effects of increasing temperatures on fish populations (Rountrey et al. 2014; Huss et al. 2019; Lindmark et al. 2022; Wootton et al. 2022). Concerns have arisen that fish body sizes will become smaller over time, thereby jeopardising yield and harvest (Daufresne et al. 2009; Baudron et al. 2014; Oke et al. 2020; Ikpewe et al. 2021). For inland recreational fisheries often managed by length‐based harvest regulations, changes in body sizes will influence the effectiveness of such regulations. However, smaller body size with warming oversimplifies the temperature size rule for species like fish that exhibit indeterminate growth (Audzijonyte et al. 2025). Documenting smaller individuals in warmer climates without evaluating patterns of growth and maturation does not provide sufficient evidence for the mechanisms driving temperature‐size rule relationships (Lindmark et al. 2023). Moreover, studies that have solely looked at changes in average body size (e.g., mean length) across temperature gradients have reached different conclusions about the direction of trends (Audzijonyte et al. 2020; Oke et al. 2020; Solokas et al. 2023). A comprehensive evaluation of the temperature‐size rule in wild fish populations is therefore needed. Understanding temperature effects on growth at multiple life stages and maturation can also inform more effective length‐based regulations, such as those designed to protect spawning fish.

Here we conduct a test of the temperature‐size rule in wild inland fish populations, using 1.4 million records of freshwater fish from 7 species in 2704 lakes, spanning 2 decades and 10 degrees of latitude in the upper Midwest United States (Frater et al. 2024). We use a Bayesian multi‐level modelling framework to fit biphasic growth models to age‐length data and water temperatures within each lake‐year‐species combination. This type of regression performed on age‐length data provides considerable information about juvenile and adult growth rates, maximum body size, and the age and length at which fish mature and begin allocating energy from somatic growth to reproduction (Honsey et al. 2017). We compared the results of this multi‐level model to a priori theoretical predictions stemming from the temperature‐size rule and its extensions. Specifically, we evaluated if warmer temperatures were associated with (a) faster juvenile growth rate, (b) earlier time to maturation, and (c) smaller size at maturation. We further quantified the effect of temperature on adult growth rates and lifespan to quantify temperature's cumulative effect on maximum body size.

## 2 Materials and Methods

### 2.1 Age‐Length Data

To test for the differences in growth patterns across temperature in freshwater fish we assembled standardised fisheries monitoring data collected by state agencies in the upper Midwest United States. The surveys were conducted between 1990 and 2021. All data in this study came specifically from lakes, so we use that specific term in place of the more general ‘waterbody’ or ‘population’ when describing methods and results. This data set contained more than 23 million fish records from standardised fishery survey data across seven states (Illinois, Indiana, Iowa, Michigan, Minnesota, South Dakota, and Wisconsin). We used paired age‐length data for seven species: Black Crappie ( *Pomoxis nigromaculatus* ), Bluegill ( *Lepomis macrochirus* ), Largemouth Bass ( *Micropterus salmoides* ), Northern Pike ( *Esox lucius* ), Smallmouth Bass ( *Micropterus dolomieu* ), Walleye ( *Sander vitreus* ), and Yellow Perch ( *Perca flavescens* ). In many cases, only a subsample of measured fish was subsequently aged, as is common in fisheries surveys. Unbiased growth estimation requires a random sample of the population; aged fish from fisheries surveys, however, do not represent a random sample because length groups are sub‐sampled without accounting for the selectivity of the survey method (Isermann and Knight 2005; Goodyear 2019). Because of this, boosting samples with age‐length keys has been shown to alleviate bias in growth data (Isermann and Knight 2005; Goodyear 2019). We used age‐length sub‐sampled data for each population to produce an age‐length key and assigned ages to the entire sample for each species, lake, and year (Frater et al. 2024; Table S1). To reduce spurious relationships stemming from small sample sizes, we only produced keys for lake‐years that had at least 5 distinct age groups with at least 5 individuals sampled in each age group. Lake‐years that did not meet these criteria were discarded, resulting in a final dataset comprising 1.4 million age‐length records from 4496 surveys across 2704 lakes.

Fish are typically aged by counting annuli on a variety of hard morphological structures (Maceina and Sammons 2006; Maceina et al. 2007). Our dataset includes fish aged using scales, otoliths, cleithra, fin rays, and spines. Although otoliths have been shown to provide the most accurate estimates of age for most fish and cleithra are the gold standard for *Esox* spp. (Beamish and McFarlane 1983; Maceina and Sammons 2006; Maceina et al. 2007), we chose to analyse all aging methods combined to increase our sample size (Table S1). We were unable to include aging method as a covariate owing to the model already being highly parameterized. However, given that there was no systematic bias in what aging methods were used during data collection, we do not believe that combining aging methods would generate spurious relationships between temperature and growth rates (Erickson 1983; Maceina and Sammons 2006; Isermann et al. 2010). Given that aging method is most likely to bias older individuals, it could be argued that the estimates of adult growth would be most sensitive to aging method; to test this, we analysed a subset of the data that only included the most reliable aging estimates (otoliths and cleithra) and found no substantial differences in the estimates of adult growth from the full model (Figures S1–S4, Table S4).

### 2.2 Temperature Data

We used modelled daily temperature profile data for lakes in the upper Midwest United States (Corson‐Dosch et al. 2023) to quantify experienced growing temperatures for each lake included in our dataset. Specifically, we used surface temperature growing‐degree days at 5°C (GDD5), which is the cumulative sum of degree‐days greater than 5°C. This serves as a metric for the total amount of thermal energy that a lake receives on an annual basis and has been widely used for studies of fish growth (Venturelli et al. 2010; Chezik et al. 2014; Hansen et al. 2017; Honsey et al. 2023). Although the choice of 5°C is somewhat arbitrary, previous work has shown that the characterisation of thermal conditions within a given year is largely insensitive to the temperature cutoff (Honsey et al. 2023) and correlates with relevant ecological metric (Flood et al. 2025; Xu et al. 2025). To capture the thermal regime experienced by each population we computed the mean GDD5 over the 10‐year period prior to and including the year in which a particular lake was sampled and refer to this quantity as $\bar{T_{10}}$. While our choice of a 10‐year timespan to characterise thermal conditions does not cover the maximum lifespan of the species in our study, it is representative of temperature regimes experienced by younger age classes (< 10 years) that make up most individuals in our dataset. Importantly, metrics calculated over different time periods are highly correlated (e.g., $\bar{T_{10}}\sim \bar{T_{5}};R_{\mathrm{adj}}^{2}=0.99$) such that choice of time periods does not alter the estimated effect of temperature.

### 2.3 Statistical Analysis

**FIGURE 1:**
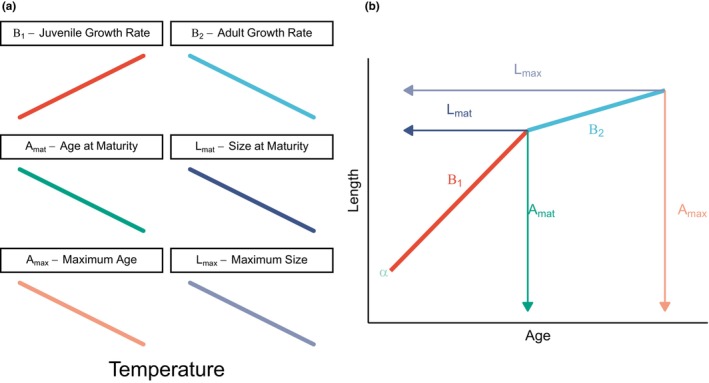
(a) Theoretical predictions from the temperature‐size rule for various growth metrics. (b) Example growth curve to show how the present analysis estimates parameters of interest to regress against temperature. Given the difficulty in interpreting *α*, we do not try to model it as a function of temperature and instead include it only as a random lake effect. Derived parameters have been placed in parentheses for clarity.

We fit a Bayesian generalised linear mixed model to age‐length data from seven freshwater fish species to evaluate the effect of temperature on growth and maturity. The model consists of a change‐point (i.e., hockey‐stick, or biphasic growth model) regression to model age‐length data of species *j* obtained during lake‐year *k* as:

$$y_{i,j,k,l}\sim \mathrm{LN}\left(\mu_{i,j,k},\sigma_{j}^{2}\right)$$

$$\mu_{i,j,k}=\left\{\begin{array}{cc} \alpha_{j,k}+\beta_{1_{j,k}}a_{i}, & a_{i}<{A_{\mathrm{mat}}}_{j,k}\\ \alpha_{j,k}+\beta_{1_{j,k}}a_{i}+\beta_{2_{j,k}}\left(a_{i}-{A_{\mathrm{mat}}}_{j,k}\right), & a_{i}\geq {A_{\mathrm{mat}}}_{j,k} \end{array}\right.$$

for i=1…n individuals, where $\mu_{i}$ is expected length, $y_{i}$ is observed length, and $a_{i}$ is age of fish i. The process error that links observed lengths to expected lengths is assumed to follow a log‐normal distribution with standard deviation $\sigma_{j}^{2}$ for j=1…J species. α, $\beta_{1}$, $\beta_{2}$, and $A_{\mathrm{mat}}$ are species‐specific parameters that describe the shape of the hockey‐stick model (Figure 1). The initial slope of the change‐point regression ($\beta_{1}$) is the first slope and represents juvenile growth rate. We expect this parameter to increase with temperature since juvenile fish are predicted to grow faster in warmer waters under the temperature‐size rule. The change‐point ($A_{\mathrm{mat}}$) is the age at which the slope transitions from $\beta_{1}$ to $\beta_{2}$ and is representative of fish transitioning from juvenile to adult growth (i.e., the age at maturity). While $A_{\mathrm{mat}}$ is not a direct measurement of sexual or reproductive maturity, this parameter of the biphasic growth model has been shown to serve as a reasonable proxy for age at maturity (Honsey et al. 2017). The temperature‐size rule predicts a lower (younger) age at maturity with increasing temperature; therefore, we predict $A_{\mathrm{mat}}$ will decrease with temperature. The temperature‐size rule also predicts a smaller size at maturity, a parameter which we can derive by combining estimates of α, $\beta_{1}$, and $A_{\mathrm{mat}}$. Lastly, $\beta_{2}$ represents the adult growth rate, which we expect to be negatively correlated with temperature as the temperature‐size rule predicts slower adult growth rates in warmer temperatures.

We model $\beta_{1}$, $A_{\mathrm{mat}}$, and $\beta_{2}$ as linear functions of historical temperature ($\bar{T_{10_{k}}}$) experienced by lake‐year *k* with random species (*j*) and lake (*l*) effects. Although α could potentially be interpreted as egg size or size of hatching (size at age zero), given that we model growth linearly, we do not believe that estimates of α, and particularly variation in α with temperature, are readily interpretable. Therefore, we model α simply as a species‐specific constant with a random lake (*l*) effect, such that:

$$\alpha_{j,k},\beta_{1_{j,k}},A_{\mathrm{ma}t_{j,k}},\beta_{2_{j,k}}\sim N\left(\varphi_{j,l,p},\sigma_{\varphi .p}^{2}\right)$$

$$\varphi_{j,k,l,p}=\left\{\begin{array}{cc} \gamma_{0}^{p}+\omega_{l}^{p}, & p=\alpha \\ \gamma_{0}^{p}+\left(\gamma_{1}^{p}\cdot \bar{T_{10_{k}}}\right)+\left(\tau_{j}^{p}\cdot \bar{T_{10_{k}}}\right)+\omega_{l}^{p}, & p=\beta_{1},A_{\mathrm{mat}},\beta_{2} \end{array}\right.$$

$$\tau_{j}^{p}\sim N\left(0,\sigma_{\tau .p}^{2}\right)$$

$$\omega_{l}^{p}\sim N\left(0,\sigma_{\omega .p}^{2}\right)$$

where $\varphi_{p}$ is the predicted value of parameter *p*, $\gamma_{0}$ and $\gamma_{1}$ are second‐level intercept and slope parameters that model the respective change‐point regression parameters against temperature and *p* indicates parameter specific coefficients. Values of each parameter (φ) are assumed to be drawn from normal distributions with standard deviation $\sigma_{\varphi }^{2}$. We included random lake intercepts ($\omega_{l}$) on all four parameters to account for random variability across space. We also included random species slopes $\left(\tau_{j}\right)$ on $\beta_{1},A_{\mathrm{mat}}$ and $\beta_{2}$ to account for taxonomic differences in sensitivity to temperature. We assumed that random effects for lakes and species are drawn from normal distributions with means 0 and standard deviations $\sigma_{\omega }^{2}$ and $\sigma_{\tau }^{2}$ respectively. While several species in our analysis exhibit sexual size dimorphism (Froese and Pauly 2025), we were unable to account for sex differences because the fish in our dataset were unsexed. Combining data across sexes may reduce precision on our estimates of average growth rates and maturation ages but should not influence our main conclusions regarding the effects of temperature.

We assigned the intercepts ($\gamma_{0}$) for each change point parameter Uniform(0, 30) priors, and the temperature‐dependent slopes of $\beta_{1},A_{\mathrm{mat}}$ and $\beta_{2}$ vague Normal(0,1) priors. Lastly, we assigned all process errors and random effect $\sigma^{2}$ parameters Gamma(1,1) priors. We derived posterior distributions of all parameters by performing Gibbs sampling using JAGS (Plummer 2003) implemented with the R2jags R package (Su and Yajima 2021) in R (R Core Team 2025). MCMC sampling was performed using 3 chains of 125,000 iterations each, a burn‐in period of 30,000, and a thinning rate of 10. We used $\hat{r}$ values to assess convergence across chains and effective sample sizes ($n_{e}$) to assess convergence within chains. We determined the statistical significance of model parameters by whether 95% highest posterior densities overlapped with zero.

To evaluate the combined impact of the various effects of temperature on maximum body size (i.e., via effects on juvenile growth rate, maturity, and adult growth rate), it is necessary to know the expected longevity of species. Temperature, however, is known to effect survival (Pauly 1980; Pepin 1991). Indeed, differential mortality has been postulated as a potential mechanism driving the temperature‐size rule in certain instances (Audzijonyte et al. 2022). To evaluate potential effects of temperature on survival, and hence lifespan, we extracted the 95th percentile age of fish in each population and fit a Bayesian generalised linear regression of 95th percentile age as a function of temperature. We assumed a Gaussian error distribution and a random effect of lake. We included the interaction between species and temperature to account for taxonomic differences in thermal sensitivity. We assigned all associated parameters vague Normal(0,10) priors. We derived posterior distributions of all parameters in R using the *brms* package (Bürkner 2017). We ran three MCMC chains for 50,000 iterations each, with a burn‐in period of 40,000, and a thinning rate of 100. We used $\hat{r}$ values to assess convergence across chains and effective sample sizes ($n_{e}$) to assess convergence within chains. All $\hat{r}$ values were less than 1.1, and all effective sample sizes were greater than 300.

To generate derived estimates of size at maturity and maximum size, we combined the parameter estimates from both analysis and modelled length at age as a function of temperature. For size at maturity, we used the posterior distributions of α, $\beta_{1}$, and $A_{\mathrm{mat}}$, and their associated temperature effects, to produce size estimates that inherit all sources of uncertainty from their constituent parts. For maximum size, we included all parameters (including temperature‐dependent maximum ages from the subsidiary analysis) to create growth curves across the entire lifespan of each species under different temperature regimes. Again, all uncertainty is carried over into these size estimates.

## 3 Results

Across the seven focal taxa, model intercepts (α) ranged from 0.07 cm (95% CI: 0.00, 0.23) to 19.66 cm (95% CI: 18.95, 20.27; Table S2). Juvenile growth rates ($\beta_{1}$) ranged from 2.63 (95% CI: 2.52, 2.66) to 11.80 (95% CI: 11.49, 12.11), and adult growth rates ($\beta_{2}$) ranged from 1.10 (95% CI: 1.02, 1.17) to 4.99 (95% CI: 4.91, 5.08). In each case the extremes were occupied by *L. macrochirus* and *E. lucius* respectively; *E. lucius* exhibiting the largest intercept, fastest juvenile growth rate, and fastest adult growth rate, whereas *L. macrochirus* exhibited the smallest intercept, slowest juvenile growth rate, and slowest adult growth rate (Table S2). The change point parameter ($A_{\mathrm{mat}}$), our proxy for age at maturity, ranged from 1.38 years (95% CI: 1.10, 1.59) to 5.36 years (95% CI: 5.26, 5.46). Although the change point parameter can be an unreliable metric in natural populations (Wootton et al. 2020), all estimates fell within the range of published values for maturation ages for each of the seven focal species (Froese and Pauly 2025).

**FIGURE 2:**
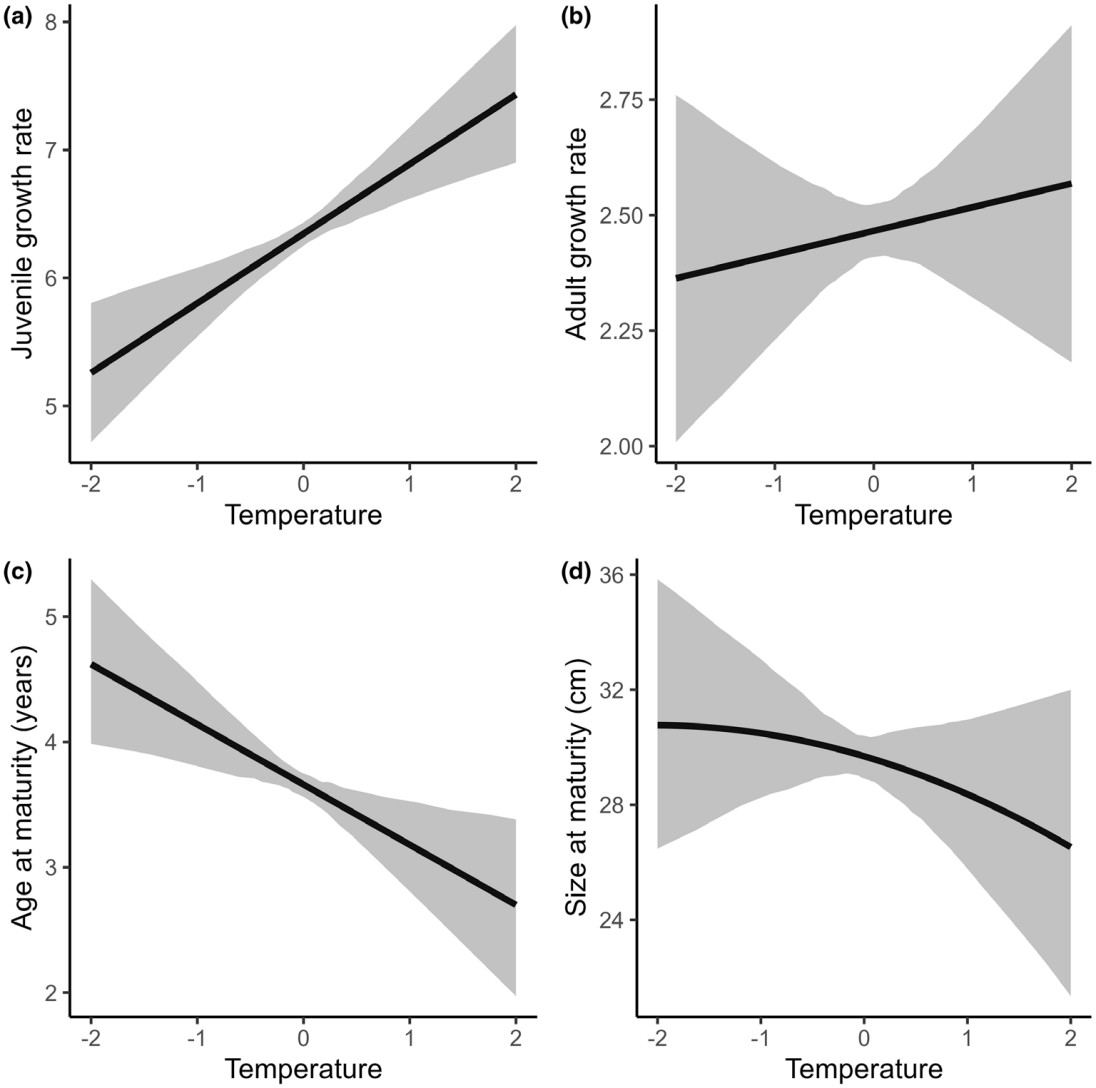
Predicted marginal effects across all species of temperature on (a) juvenile growth rate, (b) adult growth rate, (c) age at maturity, and (d) size at maturity. Solid lines reflect the mean posterior predictions and shaded areas reflect the 95% credible intervals.

**FIGURE 3:**
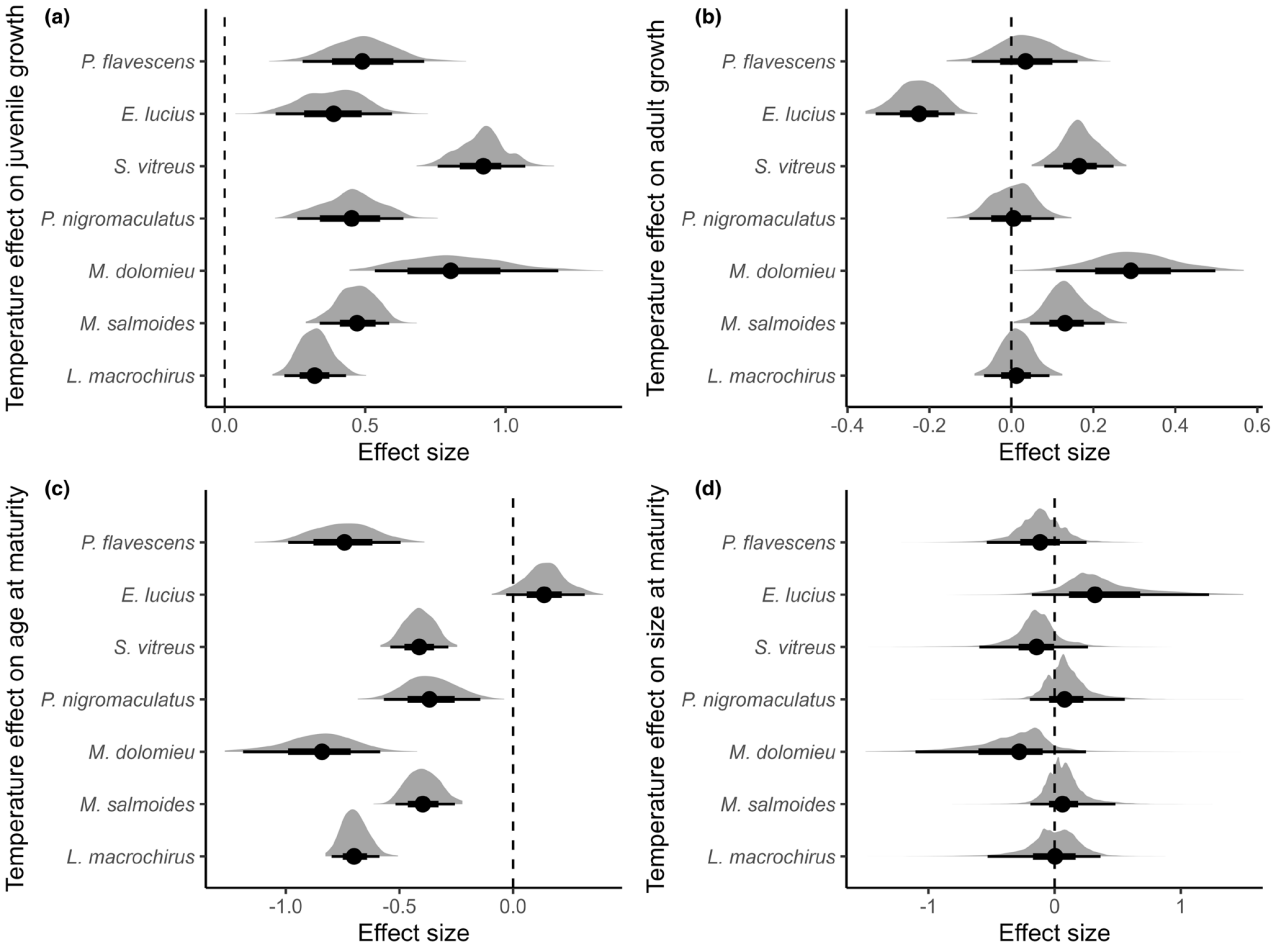
Predicted conditional (species‐specific) effects of temperature on (a) juvenile growth rate, (b) adult growth rate, and (c) age at maturity, and (d) size at maturity. Note that size at maturity is a derived parameter; as such, the posterior densities reflect first‐order derivations of the curve produced from the conditional predicted values of juvenile growth and age at maturity across temperature gradients. Points reflect median posterior estimates. Thick and thin lines denote the 66% and 95% credible intervals respectively and the shaded area shows the full posterior density. The dashed vertical lines are set at zero, indicating no effect of temperature on the parameter in question.

Across species, we found an overall positive relationship between temperature and juvenile growth rate ($\gamma_{1}^{\beta_{1}}$ = 0.54, 95% CI: 0.29, 0.80; Figure 2), and all species exhibited statistically significant positive relationships between temperature and juvenile growth rate (Figure 3, Table S3). We found an overall negative relationship between temperature and age at maturity ($\gamma_{1}^{A_{\mathrm{mat}}}$ = −0.48, 95% CI: −0.84, −0.14; Figure 2), such that fish matured on average 2 years earlier at the warmest lake‐year combinations in our study compared to the coldest ones. Within species, *E. lucius* was the only taxon that did not exhibit statistically significant negative relationships between temperature and age at maturity ( *E. lucius* , $\gamma_{1}^{A_{\mathrm{mat}}}$ = 0.14, 95% CI: −0.03, 0.32; Figure 3, Table S3). When combining estimates of juvenile growth rate and age at maturity, we found some evidence for a negative relationship between temperature and size at maturity ($\gamma_{1}^{L_{\mathrm{mat}}}$ = −0.25, 95% CI: −0.52, −0.09; Figure 2); however, when looking at the species level, we found no evidence for relationships between size at maturity and temperature (Figure 3, Table S3). Effect sizes and directions were unrelated to species thermal preferenda (Hasnain et al. 2013).

**FIGURE 4:**
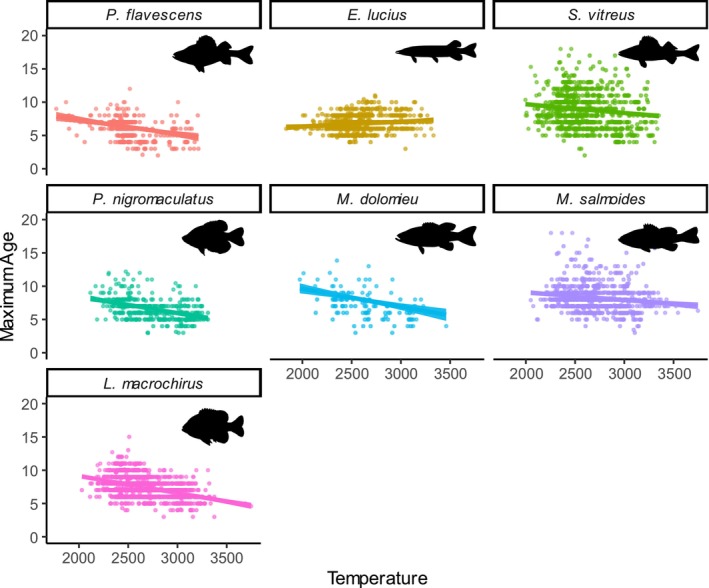
Maximum age (95th quantile) as a function of temperature (growing degree days over the previous 10 years) for seven freshwater fish species. Silhouettes reproduced without modification from PhyloPic.org under a Creative Commons licence CC0 1.0: *Esox lucius* , Timothy Knepp and Michael Keesey; *Pomoxis nigromaculatus* , Rene Martin; *Micropterus salmoides* , Carlos Cano‐Barbacil; *Lepomis macrochirus* , Corrine Avidan. Silhouettes reproduced without modification from PhyloPic.org under a Creative Commons licence CC BY‐SA 3.0 https://creativecommons.org/licenses/by‐sa/3.0/: *Perca flavescens* and *Sander vitreus* , NOAA Great Lakes Environmental Research Laboratory and Timothy Bartley; *Micropterus dolomieu* , Sherman Foote Denton and Timothy J. Bartley.

**FIGURE 5:**
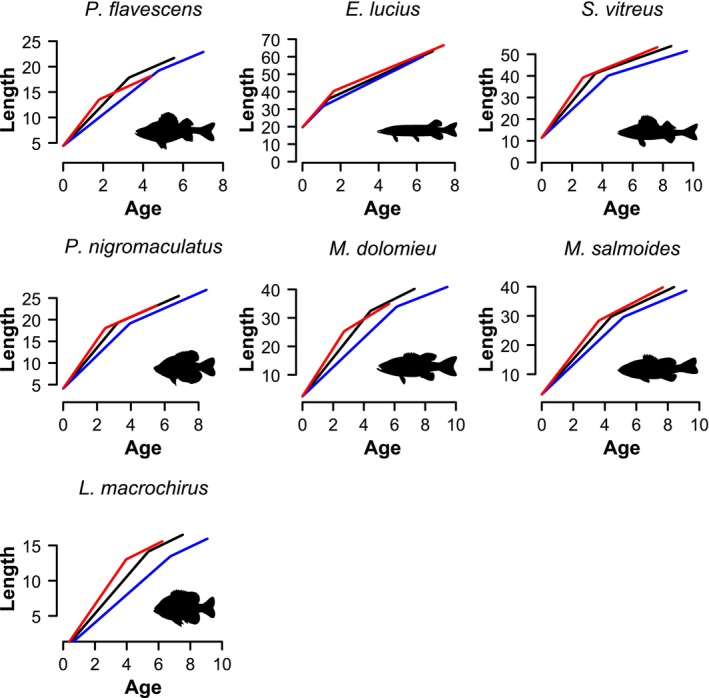
Predicted growth curves at different water temperature for all seven species. Blue lines represent the 5th percentile of $\bar{T_{10}}$ observed (i.e., coolest waters), black lines represent the median of $\bar{T_{10}}$ observed, and red lines represent the 95th percentile of $\bar{T_{10}}$ observed (i.e., warmest waters). Growth curves are projected out to the estimated longevity for each temperature regime. Silhouettes reproduced without modification from PhyloPic.org under a Creative Commons licence CC0 1.0: *Esox lucius* , Timothy Knepp and Michael Keesey; *Pomoxis nigromaculatus* , Rene Martin; *Micropterus salmoides* , Carlos Cano‐Barbacil; *Lepomis macrochirus* , Corrine Avidan. Silhouettes reproduced without modification from PhyloPic.org under a Creative Commons licence CC BY‐SA 3.0 https://creativecommons.org/licenses/by‐sa/3.0/: *Perca flavescens* and *Sander vitreus* , NOAA Great Lakes Environmental Research Laboratory and Timothy Bartley; *Micropterus dolomieu* , Sherman Foote Denton and Timothy J. Bartley.

We did not find evidence for a general relationship between temperature and adult growth rate ($\gamma_{1}^{\beta_{2}}$ = 0.05, 95% CI: −0.15, 0.23; Figure 2). Within species, however, we found evidence for positive relationships between temperature and adult growth rates in *S. vitreus* , *M. dolomieu* , and *M. salmoides* ( *S. vitreus* , $\gamma_{1}^{\beta_{2}}$ = 0.17, 95% CI: 0.08, 0.25; *M. dolomieu* , $\gamma_{1}^{\beta_{2}}$ = 0.29, 95% CI: 0.11, 0.50; *M. salmoides* , $\gamma_{1}^{\beta_{2}}$ = 0.13, 95% CI: 0.04, 0.23; Figure 3, Table S3), and a negative relationship between temperature and adult growth rates in *E. lucius* ($\gamma_{1}^{\beta_{2}}$ = −0.23, 95% CI: −0.33, −0.14; Figure 3, Table S3). From the subsidiary regression, we found evidence of a negative relationship between temperature and maximum age ($\gamma_{1}^{A_{\max}}$ = −0.37, 95% CI: −0.57, −0.15; Figure 4). Six of seven species had significant negative species‐specific relationships, whereas *E. lucius* exhibited a positive relationship between temperature and maximum age ($\gamma_{1}^{A_{\max}}$ = 0.19, 95% CI: 0.04, 0.33). As a result, most species exhibited inconsistent relationships between temperature and maximum body size (Figure 5, Table S3). The maximum size of *E. lucius* was predicted to be larger at higher temps ($\gamma_{1}^{L_{\max}}$ = 1.19, 95% CI: 0.11, 2.44; Figure 5, Table S3). Again, effect sizes and directions were unrelated to species thermal preferenda.

## 4 Discussion

The temperature‐size rule predicts that fish in warmer environments should exhibit faster juvenile growth rates, earlier and smaller maturation, and potentially smaller maximum body sizes than fish in cold environments (Atkinson 1994; Berrigan and Charnov 1994; Angilletta et al. 2004; Kuparinen et al. 2011; Crozier and Hutchings 2014). In the first empirical test of these patterns across more than a million fish in temperate lakes, we only found partial support for these expectations. Consistent with the temperature‐size rule, juvenile growth rates and maturity were faster and earlier, respectively. Inconsistent with the temperature‐size rule, we found that size at maturity remained constant with respect to temperature. In addition, maximum size did not show a consistent relationship with temperature. Therefore, while certain life‐history characteristics appear to respond to temperature as predicted, variable responses in other growth, maturation, or survival traits can yield unpredictable patterns in fish body size in response to thermal conditions.

For ectotherms, metabolic demands increase with temperature, particularly in adults (Dillon et al. 2010; Bestion et al. 2015). The ubiquity of faster juvenile growth rates, earlier maturation, and smaller maximum ages with increasing temperature is therefore consistent with life‐history theory's prediction that reduced survival selects for an accelerated pace of life (Gadgil and Bossert 1970; Reznick et al. 1990; Stearns 1992; Roff 1993; Arendt 2011). The one species with invariant maximum ages across temperature regimes, *E. lucius* , was concordantly the one species that showed invariant age at maturity across temperatures. Because we do not have data on true survival, however, we cannot rule out the possibility that faster juvenile growth rates and earlier maturity may be non‐adaptive responses to temperature (Angilletta and Dunham 2003; Gillooly et al. 2001; Kingsolver 2009; Zuo et al. 2012; Forster et al. 2012; Paaijmans et al. 2013). The assuredness of our conclusions is further challenged by the ubiquity of fishing in our study system (Xu et al. 2025). Fishing has the potential to reduce maximum age and select for faster life histories in a similar manner to the predicted effect of temperature. However, there is no correlation between temperature and fishing intensity in our study region (Deroba et al. 2007; Embke et al. 2020; Feiner et al. 2020), such that our interpretation of how temperature affects growth and maturity is not confounded.

While species overwhelmingly exhibit earlier maturation at higher temperatures, species did not exhibit a corresponding reduction in size at maturity. Size at maturity was largely invariant across temperature regimes. Optimality models assume that individuals mature at an age and size that maximises fitness; delayed maturity is a common prediction of optimality models when juvenile growth rate is low, and more time is needed to reach reproductive size (Stearns and Koella 1986; Perrin and Rubin 1990). The coupling of faster juvenile growth rate with smaller size at maturity is only expected if juvenile mortality is strongly linked to juvenile growth rate (Stearns and Koella 1986), or if asymptotic size is inversely related to juvenile growth rate (Berrigan and Charnov 1994). It is perhaps unsurprising therefore that this trend is not the norm in wild populations (Berrigan and Charnov 1994; Angilletta and Dunham 2003; Angilletta et al. 2004). Instead, we find support for plasticity in age at maturity allowing organisms to maintain a constant size at maturity, whereby an optimal reproductive schedule is achieved.

In general, adult growth rates were less affected by temperature than juvenile growth rates and higher temperatures did not result in smaller maximum body sizes. Many reasons exist for why the relationships between temperature, growth, and maturity might be different in laboratory experiments compared with a natural setting. Associations between temperature and other biotic or abiotic characteristics in the wild could obscure or even reverse the effects of temperature (Pauly 1980; Hayward and Margraf 1987; Gislason et al. 2010; Robinson et al. 2017; Salerno et al. 2021). For example, if there is a link between temperature and food availability, changes in water temperature could indirectly alter foraging behaviour and resource acquisition (Pink and Abrahams 2017; Holbrook et al. 2022). The lakes in this study vary greatly in their productivity (Heiskary and Wilson 2008); if the transient dynamics within these systems are such that the range of warmer temperatures is correlated with increased productivity, the trade‐off to allocate between somatic and reproductive growth is removed for fish in warmer, more productive systems (Hayward and Margraf 1987).

Alternatively, the failure to find clear concordance with the temperature‐size rule may stem from our overly simple metric to characterise the complex thermal regimes of lakes. In stratified lakes, fish may have seasonal access to cooler hypolimnetic waters and are thus unaffected by rising surface temperatures. However, use of these thermal refugia may itself come with a growth penalty because of lower food availability in cooler water; most of the species in our study are shallow water littoral species that occupy the epilimnion where food is most abundant. Thus, even though our temperature metric does not capture all nuances of a lake's thermal environment, it should still correlate with how a species' energy acquisition and growth will likely be affected by rising temperatures.

This study represents the most thorough test of the temperature‐size rule in wild fish populations. Previous investigations of the temperature‐size rule in wild populations only looked at changes in one component of growth, typically average or maximum body size, as it relates to temperature (Olalla‐Tárraga and Rodríguez 2007; Baudron et al. 2014; Audzijonyte et al. 2020; Oke et al. 2020; Solokas et al. 2023; Warne et al. 2024; Grabda et al. 2025). As a result, these prior studies can lend support for or against the temperature‐size rule, but do not evaluate its predictions in totality (Lindmark et al. 2023). Our results align with previous work that found equivocal support for consistent trends in body size with respect to temperature in natural systems (Huss et al. 2019; Audzijonyte et al. 2020; Lindmark et al. 2023; Solokas et al. 2023). However, it is difficult to draw comparisons with prior studies owing to the inconsistency in growth and size metrics under observation (Audzijonyte et al. 2025). Our results further reveal that temperature has the most consistent effect on juvenile growth rate and age at maturity. Only in certain instances is variation in those two traits sufficient to create hypothesised relationships between maximum size and temperature. The phenomenon of ‘shrinking’ fish is thus not necessarily a universal consequence of climate change (Audzijonyte et al. 2020; Warne et al. 2024).

Body size is intricately linked to fitness (Brown et al. 1993; Kingsolver and Huey 2008). For harvested populations specifically, body size and maturity are critical metrics for designing effective management policies. Climate‐induced changes in growth and size can therefore have profound consequences on demography and extinction risk. To accurately predict the effects of warming on wildlife, and thereby design climate‐smart mitigation strategies, we need a better understanding of how temperature shapes ontogenetic growth patterns in natural systems, not just in a laboratory setting.

## Author Contributions

All authors conceived of the study; G.C.B. and P.N.F. conducted the analyses; G.C.B., P.N.F., O.P.J., and Z.S.F. led the writing of the paper. All authors approved the manuscript.

## Conflicts of Interest

The authors declare no conflicts of interest.

## Supporting information


**Appendix S1:** ele70344‐sup‐0001‐AppendixS1.pdf.

## Data Availability

All data and code used to perform the analysis have been permanently archived on Zenodo and can be accessed at https://doi.org/10.5281/zenodo.16762147.

## References

[ele70344-bib-0001] Allen, J. A. 1877. “The Influence of Physical Conditions in the Genesis of Species.” Radical Review 1: 108–140.

[ele70344-bib-0002] Angilletta, M. J. , and A. E. Dunham . 2003. “The Temperature‐Size Rule in Ectotherms: Simple Evolutionary Explanations May Not Be General.” American Naturalist 162: 332–342. 10.1086/377187 12970841

[ele70344-bib-0003] Angilletta, M. J. , T. D. Steury , and M. W. Sears . 2004. “Temperature, Growth Rate, and Body Size in Ectotherms: Fitting Pieces of a Life‐History Puzzle.” Integrative and Comparative Biology 44: 498–509. 21676736 10.1093/icb/44.6.498

[ele70344-bib-0004] Arendt, J. D. 2011. “Size‐Fecundity Relationships, Growth Trajectories, and the Temperature‐Size Rule for Ectotherms.” Evolution 65: 43–51. 20812979 10.1111/j.1558-5646.2010.01112.x

[ele70344-bib-0005] Atkinson, D. 1994. “Temperature and Organism Size–A Biological Law for Ectotherms?” Advances in Ecological Research 25: 1–58.

[ele70344-bib-0006] Audzijonyte, A. , K. H. Andersen , D. Atkinson , et al. 2025. “Which Body Size Metrics Should Be Used for Assessing Temperature Impacts on Fish Growth and Size?” Global Change Biology 31: e70296. 40530464 10.1111/gcb.70296

[ele70344-bib-0007] Audzijonyte, A. , E. Jakubavičiūtė , M. Lindmark , and S. A. Richards . 2022. “Mechanistic Temperature‐Size Rule Explanation Should Reconcile Physiological and Mortality Responses to Temperature.” Biological Bulletin 243: 220–238. 36548974 10.1086/722027

[ele70344-bib-0008] Audzijonyte, A. , S. A. Richards , R. D. Stuart‐Smith , et al. 2020. “Fish Body Sizes Change With Temperature but Not All Species Shrink With Warming.” Nature Ecology & Evolution 4: 809–814. 32251381 10.1038/s41559-020-1171-0

[ele70344-bib-0009] Barneche, D. R. , D. R. Robertson , C. R. White , and D. J. Marshall . 2018. “Fish Reproductive‐Energy Output Increases Disproportionately With Body Size.” Science 360: 642–645. 29748282 10.1126/science.aao6868

[ele70344-bib-0010] Baudron, A. R. , C. L. Needle , A. D. Rijnsdorp , and C. T. Marshall . 2014. “Warming Temperatures and Smaller Body Sizes: Synchronous Changes in Growth of North Sea Fishes.” Global Change Biology 20: 1023–1031. 24375891 10.1111/gcb.12514

[ele70344-bib-0011] Beamish, R. J. , and G. A. McFarlane . 1983. “The Forgotten Requirement for Age Validation in Fisheries Biology.” Transactions of the American Fisheries Society 112: 735–743.

[ele70344-bib-0012] Berrigan, D. , and E. L. Charnov . 1994. “Reaction Norms for Age and Size at Maturity in Response to Temperature: A Puzzle for Life Historians.” Oikos 70: 474–478.

[ele70344-bib-0013] Bestion, E. , A. Teyssier , M. Richard , J. Clobert , and J. Cote . 2015. “Live Fast, Die Young: Experimental Evidence of Population Extinction Risk due to Climate Change.” PLoS Biology 13: e1002281. 26501958 10.1371/journal.pbio.1002281 PMC4621050

[ele70344-bib-0014] Brown, J. , J. Gillooly , A. Allen , V. Savage , and G. West . 2004. “Toward a Metabolic Theory of Ecology.” Ecology 85: 1771–1789.

[ele70344-bib-0015] Brown, J. H. , P. A. Marquet , and M. L. Taper . 1993. “Evolution of Body Size: Consequences of an Energetic Definition of Fitness.” American Naturalist 142: 573–584. 10.1086/285558 19425961

[ele70344-bib-0016] Bürkner, P. 2017. “brms: An R Package for Bayesian Multilevel Models Using Stan.” Journal of Statistical Software 80: 1–28.

[ele70344-bib-0017] Chezik, K. A. , N. P. Lester , and P. A. Venturelli . 2014. “Fish Growth and Degree‐Days I: Selecting a Base Temperature for a Within‐Population Study.” Canadian Journal of Fisheries and Aquatic Sciences 71: 47–55.

[ele70344-bib-0018] Corson‐Dosch, H. , W. Mcaliley , L. Platt , J. A. Padilla , and J. Read . 2023. Daily Water Column Temperature Predictions for Thousands of Midwest U.S. Lakes Between 1979–2022 and Under Future Climate Scenarios: U.S. Geological Survey Data Release. U.S. Geological Survey.

[ele70344-bib-0019] Crozier, L. G. , and J. A. Hutchings . 2014. “Plastic and Evolutionary Responses to Climate Change in Fish.” Evolutionary Applications 7: 68–87. 24454549 10.1111/eva.12135 PMC3894899

[ele70344-bib-0020] Daufresne, M. , K. Lengfellner , and U. Sommer . 2009. “Global Warming Benefits the Small in Aquatic Ecosystems.” Proceedings of the National Academy of Sciences of the United States of America 106: 12788–12793. 19620720 10.1073/pnas.0902080106 PMC2722360

[ele70344-bib-0021] Deroba, J. J. , M. J. Hansen , N. A. Nate , and J. M. Hennessy . 2007. “Temporal Profiles of Walleye Angling Effort, Harvest Rate, and Harvest in Northern Wisconsin Lakes.” North American Journal of Fisheries Management 27, no. 2: 717–727.

[ele70344-bib-0022] Dillon, M. E. , G. Wang , and R. B. Huey . 2010. “Global Metabolic Impacts of Recent Climate Warming.” Nature 467: 704–706. 20930843 10.1038/nature09407

[ele70344-bib-0023] Embke, H. S. , T. Beard Jr. , A. J. Lynch , and M. J. Vander Zanden . 2020. “Fishing for Food: Quantifying Recreational Fisheries Harvest in Wisconsin Lakes.” Fisheries 45, no. 12: 647–655.

[ele70344-bib-0024] Erickson, C. 1983. “Age Determination of Manitoban Walleyes Using Otoliths, Dorsal Spines, and Scales.” North American Journal of Fisheries Management 3: 176–181.

[ele70344-bib-0025] Feiner, Z. S. , M. H. Wolter , and A. W. Latzka . 2020. “‘I Will Look for You, I Will Find You, and I Will [Harvest] You’: Persistent Hyperstability in Wisconsin's Recreational Fishery.” Fisheries Research 230: 105679.

[ele70344-bib-0026] Flood, P. J. , K. E. Schiller , K. B. S. King , A. D. Runyon , K. E. Wehrly , and K. M. Alofs . 2025. “Long‐Term and Regional‐Scale Data Reveal Divergent Trends of Different Climate Variables on Fish Body Size Over 75 Years.” Global Change Biology 31: e70584. 41189541 10.1111/gcb.70584 PMC12587105

[ele70344-bib-0027] Forster, J. , A. G. Hirst , and D. Atkinson . 2012. “Warming‐Induced Reductions in Body Size Are Greater in Aquatic Than Terrestrial Species.” Proceedings of the National Academy of Sciences of the United States of America 109: 19310–19314. 23129645 10.1073/pnas.1210460109 PMC3511100

[ele70344-bib-0028] Frater, P. N. , Z. S. Feiner , G. J. Hansen , D. A. Isermann , A. W. Latzka , and O. P. Jensen . 2024. “The Incredible HALK: Borrowing Data for Age Assignment.” Fisheries 49: 117–128.

[ele70344-bib-0029] Froese, R. , and D. Pauly . 2025. “FishBase.” World Wide Web Electronic Publication. www.fishbase.org.

[ele70344-bib-0030] Gadgil, M. , and W. H. Bossert . 1970. “Life Historical Consequences of Natural Selection.” American Naturalist 104: 1–24.

[ele70344-bib-0031] Gillooly, J. F. , J. H. Brown , G. B. West , V. M. Savage , and E. L. Charnov . 2001. “Effects of Size and Temperature on Metabolic Rate.” Science 293: 2248–2251. 11567137 10.1126/science.1061967

[ele70344-bib-0032] Gislason, H. , N. Daan , J. C. Rice , and J. G. Pope . 2010. “Size, Growth, Temperature and the Natural Mortality of Marine Fish.” Fish and Fisheries 11: 149–158.

[ele70344-bib-0033] Goodyear, C. P. 2019. “Modeling Growth: Consequences From Selecting Samples by Size.” Transactions of the American Fisheries Society 148: 528–551.

[ele70344-bib-0034] Grabda, E. E. , P. J. Flood , K. B. King , J. Breck , K. E. Wehrly , and K. Alofs . 2025. “Mismatch Between Climate‐Based Bioenergetics Model of Fish Growth and Long‐Term and Regional‐Scale Empirical Data.” Canadian Journal of Fisheries and Aquatic Sciences 82: 1–15.

[ele70344-bib-0035] Hansen, G. J. , J. S. Read , J. F. Hansen , and L. A. Winslow . 2017. “Projected Shifts in Fish Species Dominance in Wisconsin Lakes Under Climate Change.” Global Change Biology 23, no. 4: 1463–1476. 27608297 10.1111/gcb.13462

[ele70344-bib-0036] Hasnain, S. S. , B. J. Shuter , and C. K. Minns . 2013. “Phylogeny Influences the Relationships Linking Key Ecological Thermal Metrics for North American Freshwater Fish Species.” Canadian Journal of Fisheries and Aquatic Sciences 70: 964–972.

[ele70344-bib-0037] Hayward, R. S. , and F. J. Margraf . 1987. “Eutrophication Effects on Prey Size and Food Available to Yellow Perch in Lake Erie.” Transactions of the American Fisheries Society 116: 210–223.

[ele70344-bib-0038] Heiskary, S. , and B. Wilson . 2008. “Minnesota's Approach to Lake Nutrient Criteria Development.” Lake and Reservoir Management 24: 282–297.

[ele70344-bib-0039] Holbrook, B. V. , B. J. Bethke , M. D. Bacigalupi , and D. F. Staples . 2022. “Assessing Minnesota's Changing Yellow Perch Populations Using Length‐Based Metrics.” North American Journal of Fisheries Management 42: 642–658.

[ele70344-bib-0040] Honsey, A. D. , A. L. Rypel , and P. A. Venturelli . 2023. “Guidance for Selecting Base Temperatures When Using Degree‐Days in Fish Growth Analyses.” Canadian Journal of Fisheries and Aquatic Sciences 80: 549–562.

[ele70344-bib-0041] Honsey, A. E. , D. F. Staples , and P. A. Venturelli . 2017. “Accurate Estimates of Age at Maturity From the Growth Trajectories of Fishes and Other Ectotherms.” Ecological Applications 27: 182–192. 27973729 10.1002/eap.1421

[ele70344-bib-0042] Huss, M. , M. Lindmark , P. Jacobson , R. M. van Dorst , and A. Gårdmark . 2019. “Experimental Evidence of Gradual Size‐Dependent Shifts in Body Size and Growth of Fish in Response to Warming.” Global Change Biology 25: 2285–2295. 30932292 10.1111/gcb.14637 PMC6850025

[ele70344-bib-0043] Ikpewe, I. E. , A. R. Baudron , A. Ponchon , and P. G. Fernandes . 2021. “Bigger Juveniles and Smaller Adults: Changes in Fish Size Correlate With Warming Seas.” Journal of Applied Ecology 58: 847–856.

[ele70344-bib-0044] Isermann, D. A. , and C. T. Knight . 2005. “A Computer Program for Age–Length Keys Incorporating Age Assignment to Individual Fish.” North American Journal of Fisheries Management 25: 1153–1160.

[ele70344-bib-0045] Isermann, D. A. , M. H. Wolter , and J. J. Breeggemann . 2010. “Estimating Black Crappie Age: An Assessment of Dorsal Spines and Scales as Nonlethal Alternatives to Otoliths.” North American Journal of Fisheries Management 30: 1591–1598.

[ele70344-bib-0048] Kingsolver, J. , and R. Huey . 2008. “Size, Temperature, and Fitness: Three Rules.” Evolutionary Ecology Research 10: 251–268.

[ele70344-bib-0049] Kingsolver, J. G. 2009. “The Well‐Temperatured Biologist: American Society of Naturalists Presidential Address.” American Naturalist 174: 755–768. 10.1086/648310 19857158

[ele70344-bib-0050] Kuparinen, A. , J. M. Cano , J. Loehr , G. Herczeg , A. Gonda , and J. Merilä . 2011. “Fish Age at Maturation Is Influenced by Temperature Independently of Growth.” Oecologia 167: 435–443. 21479961 10.1007/s00442-011-1989-x

[ele70344-bib-0051] Lawson, Z. J. , and S. R. Carpenter . 2014. “A Morphometric Approach for Stocking Walleye Fingerlings in Lakes Invaded by Rainbow Smelt.” North American Journal of Fisheries Management 34: 998–1002.

[ele70344-bib-0052] Lindmark, M. , A. Audzijonyte , J. L. Blanchard , and A. Gårdmark . 2022. “Temperature Impacts on Fish Physiology and Resource Abundance Lead to Faster Growth but Smaller Fish Sizes and Yields Under Warming.” Global Change Biology 28: 6239–6253. 35822557 10.1111/gcb.16341 PMC9804230

[ele70344-bib-0053] Lindmark, M. , M. Karlsson , and A. Gårdmark . 2023. “Larger but Younger Fish When Growth Outpaces Mortality in Heated Ecosystem.” eLife 12: e82996. 37157843 10.7554/eLife.82996 PMC10168697

[ele70344-bib-0054] Maceina, M. , and S. Sammons . 2006. “An Evaluation of Different Structures to Age Freshwater Fish From a Northeastern US River.” Fisheries Management and Ecology 13: 237–242.

[ele70344-bib-0055] Maceina, M. J. , J. Boxrucker , D. L. Buckmeier , et al. 2007. “Current Status and Review of Freshwater Fish Aging Procedures Used by State and Provincial Fisheries Agencies With Recommendations for Future Directions.” Fisheries 32: 329–340.

[ele70344-bib-0056] Oke, K. B. , C. J. Cunningham , P. A. H. Westley , et al. 2020. “Recent Declines in Salmon Body Size Impact Ecosystems and Fisheries.” Nature Communications 11: 4155. 10.1038/s41467-020-17726-z PMC7438488 32814776

[ele70344-bib-0057] Olalla‐Tárraga, M. Á. , and M. Á. Rodríguez . 2007. “Energy and Interspecific Body Size Patterns of Amphibian Faunas in Europe and North America: Anurans Follow Bergmann's Rule, Urodeles Its Converse.” Global Ecology and Biogeography 16: 606–617.

[ele70344-bib-0058] Paaijmans, K. P. , R. L. Heinig , R. A. Seliga , et al. 2013. “Temperature Variation Makes Ectotherms More Sensitive to Climate Change.” Global Change Biology 19: 2373–2380. 23630036 10.1111/gcb.12240 PMC3908367

[ele70344-bib-0059] Pauly, D. 1980. “On the Interrelationships Between Natural Mortality, Growth Parameters, and Mean Environmental Temperature in 175 Fish Stocks.” ICES Journal of Marine Science 39: 175–192.

[ele70344-bib-0060] Pepin, P. 1991. “Effect of Temperature and Size on Development, Mortality, and Survival Rates of the Pelagic Early Life History Stages of Marine Fish.” Canadian Journal of Fisheries and Aquatic Sciences 48: 503–518.

[ele70344-bib-0061] Perrin, N. , and J. F. Rubin . 1990. “On Dome‐Shaped Norms of Reaction for Size‐to‐Age at Maturity in Fishes.” Functional Ecology 4: 53–57.

[ele70344-bib-0062] Pink, M. , and M. V. Abrahams . 2017. “Temperature and Its Impact on Predation Risk Within Aquatic Ecosystems.” Canadian Journal of Fisheries and Aquatic Sciences 73: 869–876.

[ele70344-bib-0063] Plummer, M. 2003. “JAGS: A Program for Analysis of Bayesian Graphical Models Using Gibbs Sampling.” Proceedings of the 3rd International Workshop on Distributed Statistical Computing, Vienna, Austria, 1–10.

[ele70344-bib-0064] R Core Team . 2025. R: A Language and Environment for Statistical Computing. R Foundation for Statistical Computing.

[ele70344-bib-0065] Ray, C. 1960. “The Application of Bergmann's and Allen's Rules to the Poikilotherms.” Journal of Morphology 106: 85–108. 14436612 10.1002/jmor.1051060104

[ele70344-bib-0066] Reznick, D. A. , H. Bryga , and J. A. Endler . 1990. “Experimentally Induced Life‐History Evolution in a Natural Population.” Nature 346: 357–359.

[ele70344-bib-0067] Robinson, J. P. , I. D. Williams , A. M. Edwards , et al. 2017. “Fishing Degrades Size Structure of Coral Reef Fish Communities.” Global Change Biology 23: 1009–1022. 27564866 10.1111/gcb.13482

[ele70344-bib-0068] Roff, D. 1993. Evolution of Life Histories: Theory and Analysis. Springer Science & Business Media.

[ele70344-bib-0069] Rountrey, A. N. , P. G. Coulson , J. J. Meeuwig , and M. Meekan . 2014. “Water Temperature and Fish Growth: Otoliths Predict Growth Patterns of a Marine Fish in a Changing Climate.” Global Change Biology 20: 2450–2458. 24862838 10.1111/gcb.12617

[ele70344-bib-0070] Salerno, M. , M. Berlino , M. C. Mangano , and G. Sarà . 2021. “Microplastics and the Functional Traits of Fishes: A Global Meta‐Analysis.” Global Change Biology 27: 2645–2655. 33638211 10.1111/gcb.15570

[ele70344-bib-0071] Solokas, M. A. , Z. S. Feiner , R. Al‐Chokachy , et al. 2023. “Shrinking Body Size and Climate Warming: Many Freshwater Salmonids Do Not Follow the Rule.” Global Change Biology 29: 2478–2492. 36734695 10.1111/gcb.16626

[ele70344-bib-0072] Stearns, S. C. 1992. The Evolution of Life Histories. Oxford University Press.

[ele70344-bib-0073] Stearns, S. C. , and J. C. Koella . 1986. “The Evolution of Phenotypic Plasticity in Life‐History Traits: Predictions of Reaction Norms for Age and Size at Maturity.” Evolution 40: 893–913. 28556219 10.1111/j.1558-5646.1986.tb00560.x

[ele70344-bib-0074] Su, Y.‐S. , and M. Yajima . 2021. R2jags: Using R to Run ‘JAGS’. R package version 0.7‐1. https://CRAN.R‐project.org/package=R2jags.

[ele70344-bib-0075] Venturelli, P. A. , N. P. Lester , T. R. Marshall , and B. J. Shuter . 2010. “Consistent Patterns of Maturity and Density‐Dependent Growth Among Populations of Walleye (*Sander vitreus*): Application of the Growing Degree‐Day Metric.” Canadian Journal of Fisheries and Aquatic Sciences 67: 1057–1067.

[ele70344-bib-0077] Warne, C. P. , M. M. Guzzo , K. Cazelles , C. Chu , N. Rooney , and K. S. McCann . 2024. “Thermal Tolerance and Habitat Preferences Mediate How Freshwater Fish Body Sizes Respond to Warming.” Canadian Journal of Fisheries and Aquatic Sciences 81: 488–496.

[ele70344-bib-0078] White, E. P. , S. M. Ernest , A. J. Kerkhoff , and B. J. Enquist . 2007. “Relationships Between Body Size and Abundance in Ecology.” Trends in Ecology & Evolution 22: 323–330. 17399851 10.1016/j.tree.2007.03.007

[ele70344-bib-0079] Wootton, H. F. , J. R. Morrongiello , and A. Audzijonyte . 2020. “Estimating Maturity From Size‐At‐Age Data: Are Real‐World Fisheries Datasets up to the Task?” Reviews in Fish Biology and Fisheries 30: 681–697.

[ele70344-bib-0080] Wootton, H. F. , J. R. Morrongiello , T. Schmitt , and A. Audzijonyte . 2022. “Smaller Adult Fish Size in Warmer Water Is Not Explained by Elevated Metabolism.” Ecology Letters 25: 1177–1188. 35266600 10.1111/ele.13989 PMC9545254

[ele70344-bib-0081] Xu, L. , H. S. Embke , C. M. Free , et al. 2025. “Disentangling the Historical Impacts of Warming and Fishing on Exploited Freshwater Fish Populations.” Science Advances 11: eadx5138. 41032593 10.1126/sciadv.adx5138 PMC12487873

[ele70344-bib-0082] Zuo, W. , M. E. Moses , G. B. West , C. Hou , and J. H. Brown . 2012. “A General Model for Effects of Temperature on Ectotherm Ontogenetic Growth and Development.” Proceedings of the Royal Society B: Biological Sciences 279: 1840–1846. 10.1098/rspb.2011.2000 PMC3297449 22130604

