# Supplementary material for: Mixed Support for the Temperature‐Size Rule in Wild Freshwater Fishes
Source: Ecol Lett. 2026 Feb 18;29(2):e70344. doi: 10.1111/ele.70344 (PMC12916084; doi:10.1111/ele.70344)
Supplement: Supplementary file 1 — Appendix S1: ele70344‐sup‐0001‐AppendixS1.pdf. [file ELE-29-0-s001.pdf]

Supplementary material for: Mixed support for the temperature-size rule in wild freshwater fishes

George C. Brooks[1], Paul N. Frater[1], Olaf P. Jensen[1], Gretchen J.A. Hansen[2], Craig Paukert[3], Michael Verhoeven[2], Lyndsie Wszola[3], Luoliang Xu[1], Zachary S. Feiner[1,4]

[1]Center for Limnology, University of Wisconsin-Madison, Madison, WI, USA

[2]Department of Fisheries, Wildlife and Conservation Biology, University of Minnesota, St. Paul, MN, USA

[3]U.S. Geological Survey, Missouri Cooperative Fish and Wildlife Research Unit, School of Natural Resources, University of Missouri, Columbia, MO, USA

[4]Office of Applied Science, Wisconsin Department of Natural Resources, Science Operations Center, Madison, Wisconsin, USA

Fish are typically aged by counting annuli on a variety of morphological structures. Our dataset included fish aged using scales, otoliths, cleithra, fin rays, and spines. Among these, otoliths have been shown to provide the most accurate estimates of age for most fish and cleithera are the standard for *Esox* spp. (Maceina and Sammons 2006, Maceina et al. 2007). In the main text for this article, we chose to analyze all aging methods combined to increase our sample size (Table S1). However, if certain aging estimates are inaccurate, combining aging methods may generate spurious relationships between temperature and growth rates. To alleviate these concerns, here we reproduce the analysis presented in the paper using only otolith and cleithera data. Table S4 is the equivalent of Table S3 but based on the data subset, and Figures S1-S4 are the equivalent of Figures 2-5 in the main text but with the data subset.

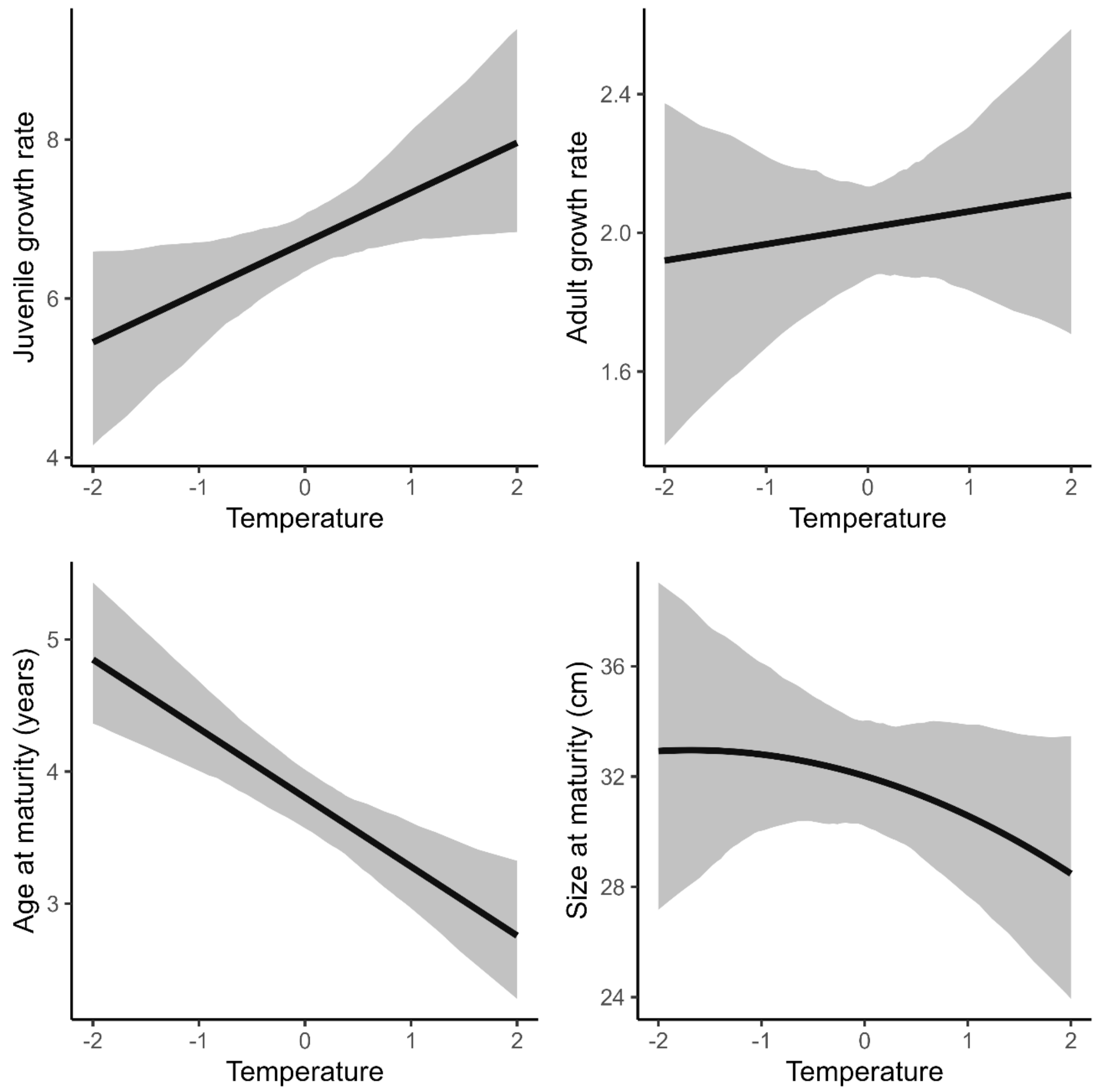

Figure S1. Predicted marginal effects of temperature on a) juvenile growth rate, b) adult growth rate, c) age at maturity, and d) size at maturity. Solid lines reflect the mean posterior predictions and shaded areas reflect the 95% credible intervals. Parameter estimates are obtained using a subset of the data that only includes the most reliable aging method (otoliths and cleithera).

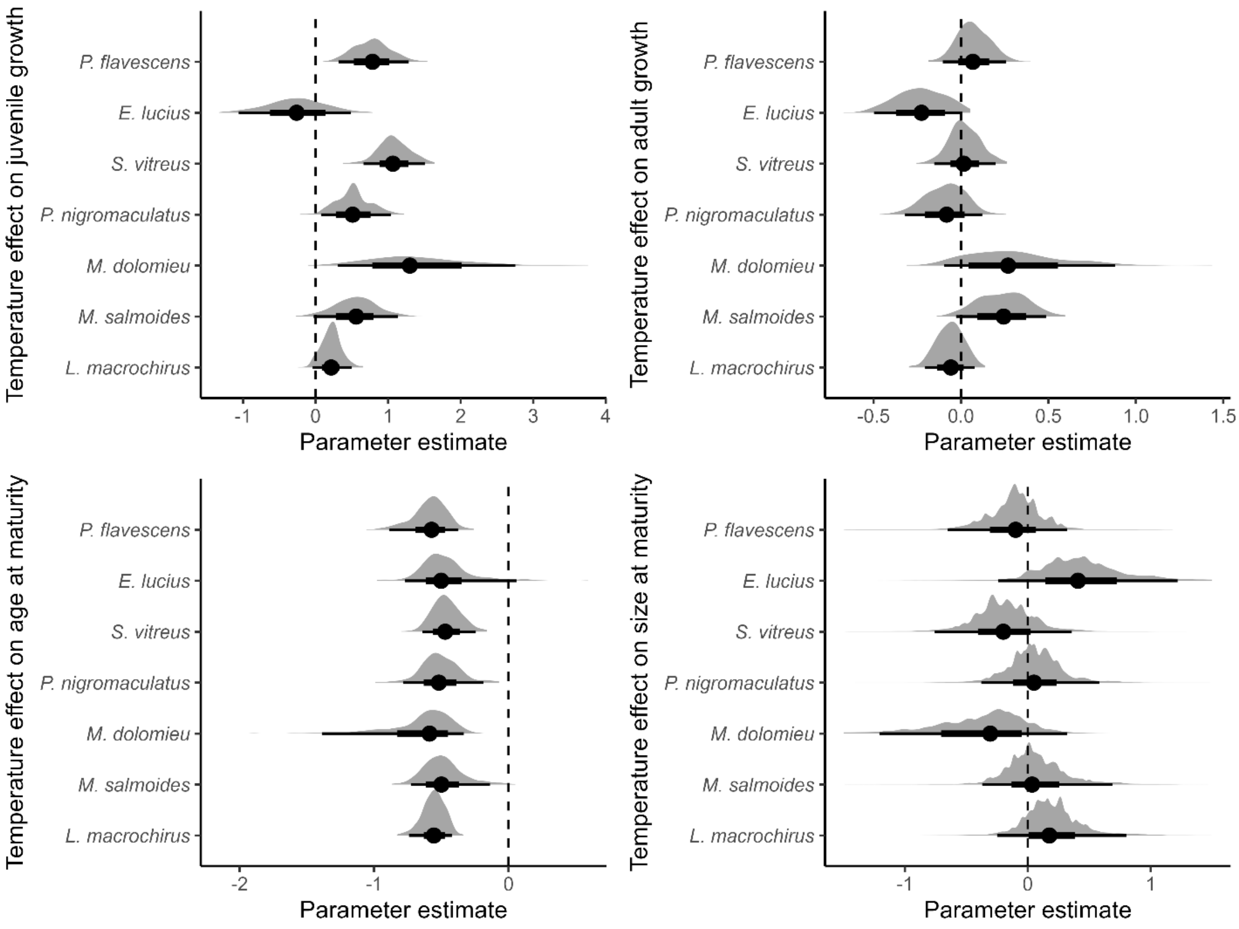

Figure S2. Predicted conditional (species-specific) effects of temperature on a) juvenile growth rate, b) adult growth rate, and c) age at maturity, and d) size at maturity. Note that size at maturity is a derived parameter; as such, the posterior densities reflect first-order derivations of the curve produced from the conditional predicted values of juvenile growth and age at maturity across temperature gradients. Points reflect median posterior estimates. Thick and thin lines denote the 66% and 95% credible intervals respectively. The shaded area shows the full posterior density. Parameter estimates are obtained using a subset of the data that only includes the most reliable aging method (otoliths and cleithera).

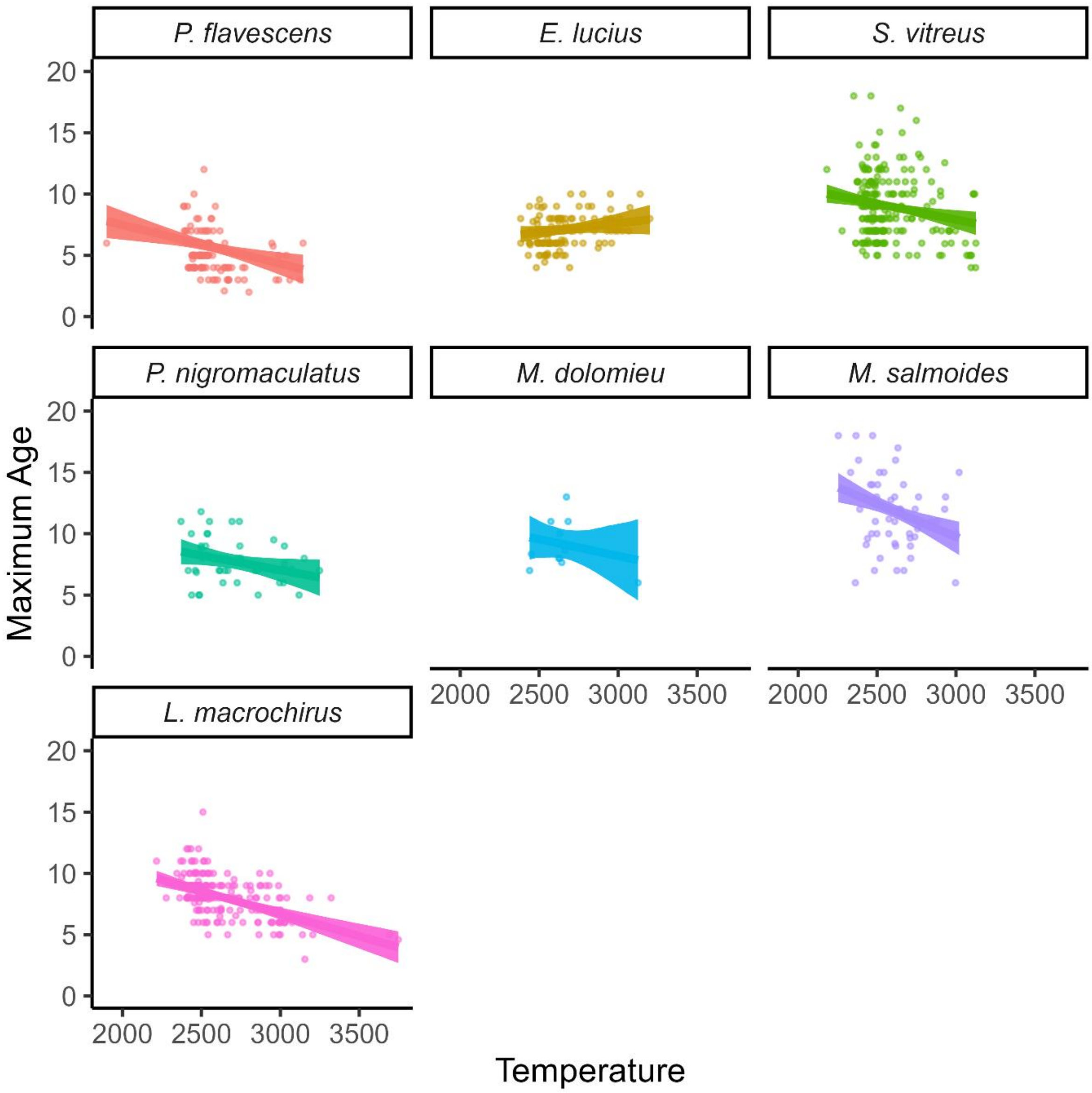

Figure S3. Maximum age (95th quantile) as a function of temperature for seven freshwater fish species. Parameter estimates are obtained using a subset of the data that only includes the most reliable aging method (otoliths and cleithera).

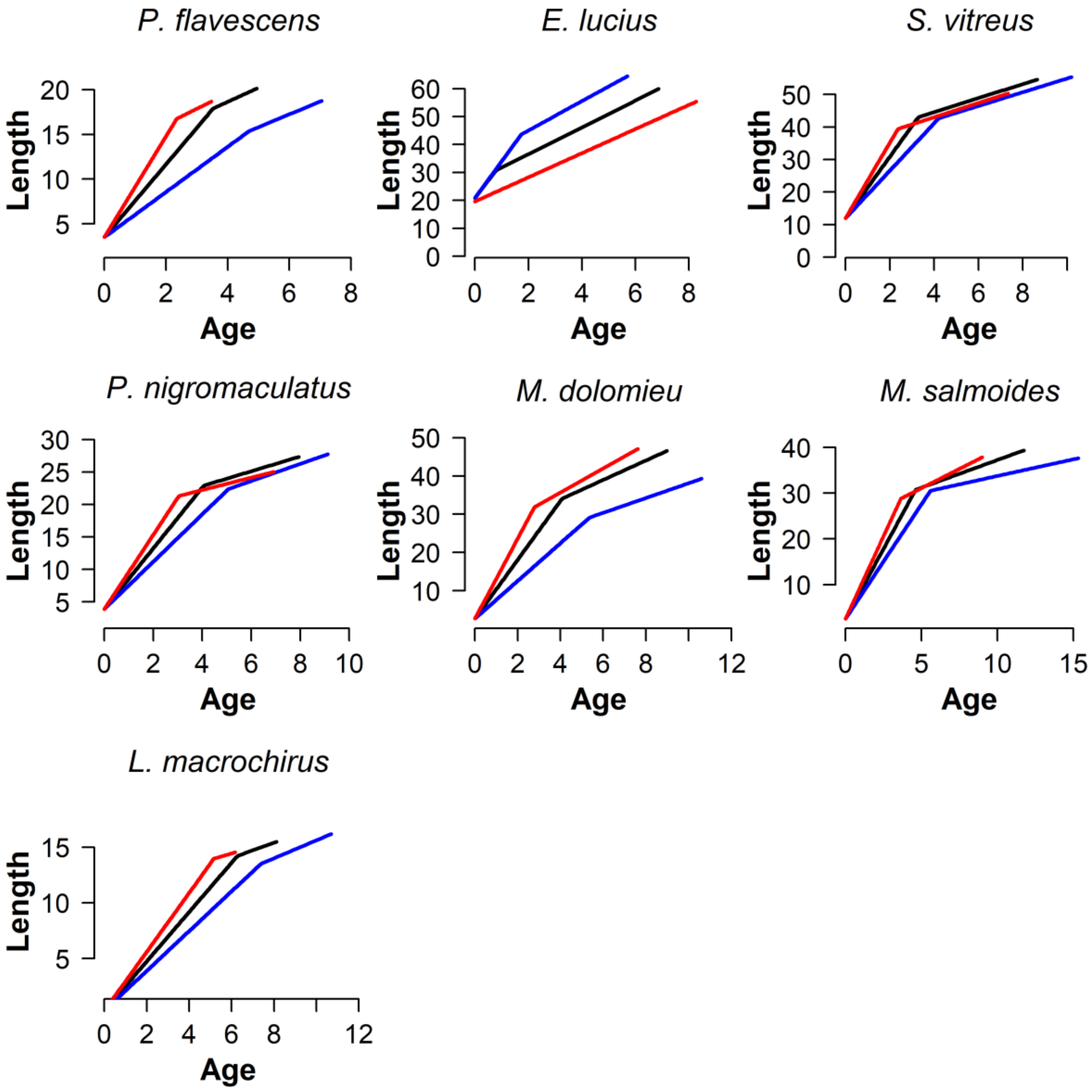

Figure S4. Predicted growth curves at different water temperature for all seven species. Blue lines represent the 5th percentile of $\overline{T_{10}}$ observed (i.e. coolest waters), black lines represent the median of $\overline{T_{10}}$ observed, and red lines represent the 95th percentile of $\overline{T_{10}}$ observed (i.e. warmest waters). Growth curves are projected out to the estimated longevity for each temperature regime. Parameter estimates are obtained using a subset of the data that only includes the most reliable aging method (otoliths).

Table S1. Age-length data coverage by aging method and species. Numbers in parentheses represent number of aged fish and all other values represent sample sizes including inferred ages obtained using age-length keys (Frater et al. 2024).

	**scales**	**otoliths**	**spines**	**fin rays**	**cleithra**	**combined**
**Coolwater**						
P. flavescens	76,967 (12,552)	117,596 (21,620)	1,477 (488)	1,428 (211)	0 (0)	197,468 (34,871)
E. lucius	107,282 (62,009)	216 (184)	1,743 (1,215)	21,203 (7,780)	16,671 (11,950)	147,115 (83,138)
S. vitreus	100,194 (51,250)	89,741 (45,834)	132,086 (22,811)	0 (0)	0 (0)	322,021 (119,895)
**Warmwater**						
P. nigromaculatus	89,680 (22,946)	11,786 (4,003)	422 (256)	200 (71)	0 (0)	102,088 (27,276)
M. dolomieu	23,415 (10,525)	2,395 (1,134)	991 (477)	0 (0)	0 (0)	26,801 (12,136)
M. salmoides	88,652 (46,006)	11,539 (5,302)	21,777 (5,808)	0 (0)	0 (0)	121,968 (57,116)
L. macrochirus	413,923 (89,541)	76,354 (20,237)	6,035 (732)	0 (0)	0 (0)	496,312 (110,510)
**Total**	900,113 (294,829)	308,751 (98,314)	164,531 (31,787)	22,831 (8,062)	16,671 (11,950)	1,413,773 (444,942)

Table S2. Species-specific growth parameters. Parameter estimates are conditional on temperature. Species are ordered by thermal optima (Final Temperature Preferenda; Hasnain et al. 2013) from low to high and grouped by thermal guild. Values represent posterior medians with 95% credible intervals in parentheses.

	$\boldsymbol{\alpha}$<br>**length at age-0 (cm)**	$\boldsymbol{\beta_1}$<br>**juvenile growth rate (cm/yr)**	$\boldsymbol{\beta_2}$<br>**adult growth rate (cm/yr)**	$\boldsymbol{A_{mat}}$<br>**age at maturity (years)**
Coolwater				
P. flavescens	4.41 (3.60, 5.42)	4.08 (3.77, 4.38)	1.71 (1.54, 1.90)	3.29 (2.97, 3.61)
E. lucius	19.66 (18.95, 20.27)	11.80 (11.49, 12.11)	4.99 (4.91, 5.08)	1.38 (1.10, 1.59)
S. vitreus	11.42 (10.96, 11.88)	8.39 (8.22, 8.56)	2.51 (2.43, 2.61)	3.55 (3.39, 3.68)
Warmwater				
P. nigromaculatus	4.10 (3.30, 4.83)	4.71 (4.48, 4.93)	1.72 (1.59, 1.86)	3.22 (2.93, 3.47)
M. dolomieu	2.45 (0.90, 3.94)	6.77 (6.36, 7.19)	2.69 (2.42, 2.96)	4.43 (4.08, 4.78)
M. salmoides	3.11 (2.53, 3.67)	6.04 (5.90, 6.20)	2.54 (2.43, 2.64)	4.41 (4.24, 4.55)
L. macrochirus	0.07 (0.01, 0.23)	2.63 (2.52, 2.72)	1.10 (1.02, 1.17)	5.36 (5.26, 5.46)

Table S3. Species-specific relationships between temperature and growth parameters. Species are ordered by thermal optima (Final Temperature Preferenda; Hasnain et al. 2013) from low to high and grouped by thermal guild. Values represent posterior medians with 95% credible intervals in parentheses. Note that size at maturity and maximum size are derived parameters; as such, the posterior densities reflect first-order derivations of the curve produced from the conditional predicted values of juvenile growth and age at maturity across temperature gradients. Estimates for which the 95% credible intervals do not overlap zero are highlighted in bold.

	Juvenile Growth	***Age at Maturity***	***Size at Maturity***	***Adult Growth***	***Maximum Age***	***Maximum size***
Coolwater						
P. flavescens	**0.49 (0.28, 0.71)**	**-0.74 (-0.99, -0.49)**	-0.12 (-0.54, 0.25)	0.03 (-0.10, 0.16)	**-0.57 (-0.79, -0.36)**	-1.05 (-2.91, 0.85)
E. lucius	**0.39 (0.18, 0.60)**	0.14 (-0.03, 0.32)	0.32 (-0.18, 1.40)	**-0.23 (-0.33, -0.14)**	**0.19 (0.06, 0.33)**	**1.19 (0.11, 2.44)**
S. vitreus	**0.92 (0.76, 1.07)**	**-0.41 (-0.54, -0.29)**	-0.14 (-0.60, 0.26)	**0.17 (0.08, 0.25)**	**-0.35 (-0.48, -0.22)**	0.84 (-1.05, 2.62)
Warmwater						
P. nigromaculatus	**0.45 (0.26, 0.64)**	**-0.37 (-0.57, -0.14)**	0.08 (-0.20, 0.56)	0.01 (-0.10, 0.10)	**-0.67 (-0.85, -0.50)**	-0.79 (-1.90, 0.41)
M. dolomieu	**0.80 (0.53, 1.19)**	**-0.84 (-1.19, -0.58)**	-0.28 (-1.21, 0.25)	**0.29 (0.11, 0.50)**	**-0.71 (-1.00, -0.43)**	-0.81 (-3.95, 2.93)
M. salmoides	**0.47 (0.34, 0.59)**	**-0.40 (-0.52, -0.26)**	0.06 (-0.19, 0.48)	**0.13 (0.04, 0.23)**	**-0.31 (-0.44, -0.18)**	0.41 (-0.64, 1.54)
L. macrochirus	**0.32 (0.21, 0.43)**	**-0.70 (-0.80, -0.59)**	0.00 (-0.53, 0.36)	0.01 (-0.07, 0.09)	**-0.68 (-0.79, -0.58)**	-0.11 (-1.07, 1.18)

Table S4. Species-specific relationships between temperature and growth parameters. Estimates presented here were obtained from modelling a subset of the age-length data that contained the most reliable ageing method (otoliths and cleithera). Species are ordered by thermal optima (Final Temperature Preferenda; Hasnain et al. 2013) from low to high and grouped by thermal guild. Values represent posterior medians with 95% credible intervals in parentheses. Note that size at maturity and maximum size are derived parameters; as such, the posterior densities reflect first-order derivations of the curve produced from the conditional predicted values of juvenile growth and age at maturity across temperature gradients. Estimates for which the 95% credible intervals do not overlap zero are highlighted in bold

Species	***Juvenile Growth***	***Age at Maturity***	***Size at Maturity***	***Adult Growth***	***Maximum Age***	***Maximum size***
Coolwater						
P. flavescens	**0.78 (0.32, 1.28)**	**-0.57 (-0.89, -0.37)**	-0.10 (-0.68, 0.32)	0.07 (-0.10, 0.26)	**-0.66 (-1.07, -0.25)**	1.89 (-0.43, 5.09)
E. lucius	-0.26 (-1.06, 0.49)	-0.50 (-0.77, 0.06)	0.42 (-0.31, 1.97)	-0.23 (-0.50, 0.00)	0.32 (-0.13, 0.76)	**-7.97 (-11.5, -4.00)**
S. vitreus	**1.07 (0.66, 1.51)**	**-0.47 (-0.64, -0.24)**	-0.20 (-0.76, 0.36)	0.02 (-0.15, 0.20)	**-0.54 (-0.89, -0.20)**	-0.88 (-3.85, 2.21)
Warmwater						
P. nigromaculatus	**0.51 (0.07, 1.04)**	**-0.52 (-0.78, -0.19)**	0.05 (-0.38, 0.60)	-0.08 (-0.32, 0.12)	-0.50 (-1.04, 0.00)	-1.82 (-4.67, 2.03)
M. dolomieu	**1.30 (0.31, 2.75)**	**-0.59 (-1.39, -0.33)**	-0.33 (-2.35, 0.45)	0.27 (-0.10, 0.88)	-0.58 (-1.93, 0.77)	0.52 (-5.32, 10.4)
M. salmoides	0.56 (-0.03, 1.14)	**-0.50 (-0.72, -0.13)**	0.03 (-0.38, 0.69)	0.24 (-0.03, 0.49)	**-1.14 (-1.76, -0.51)**	0.45 (-2.70, 4.39)
L. macrochirus	0.21 (-0.04, 0.50)	**-0.56 (-0.74, -0.42)**	0.18 (-0.25, 0.82)	-0.06 (-0.21, 0.08)	**-0.77 (-1.02, -0.53)**	-0.21 (-1.85, 1.95)

Literature Cited

Frater, P.N., Feiner, Z.S., Hansen, G.J., Isermann, D.A., Latzka, A.W. and Jensen, O.P., 2024. The Incredible HALK: Borrowing Data for Age Assignment. Fisheries, 49(3), pp.117-128.

Hasnain, S.S., Shuter, B.J. and Minns, C.K., 2013. Phylogeny influences the relationships linking key ecological thermal metrics for North American freshwater fish species. Canadian Journal of Fisheries and Aquatic Sciences, 70(7), pp.964-972.

Maceina, M., and S. Sammons. 2006. An evaluation of different structures to age freshwater fish from a northeastern US river. Fisheries Management and Ecology 13(4):237–242.

Maceina, M.J., Boxrucker, J., Buckmeier, D.L., Gangl, R.S., Lucchesi, D.O., Isermann, D.A., Jackson, J.R. and Martinez, P.J., 2007. Current status and review of freshwater fish aging procedures used by state and provincial fisheries agencies with recommendations for future directions. Fisheries, 32(7), pp.329-340.
